# Long-Term Clinical Outcomes of PCI Versus Medical Therapy in NSTEMI Patients with Prior CABG

**DOI:** 10.3390/medicina62020315

**Published:** 2026-02-03

**Authors:** Onur Altınkaya, Selim Aydemir, Murat Özmen, Mustafa Özkoç, Rauf Macit, Abuzer Ocak, Emrah Aksakal

**Affiliations:** 1 Department of Cardiology, University of Health Sciences, Erzurum City Hospital, 25240 Erzurum, Turkey; selim1723@hotmail.com (S.A.); drmuratt1987@gmail.com (M.Ö.); md.mustafaozkoc@gmail.com (M.Ö.); emrahaksakal@gmail.com (E.A.); 2 Department of Cardiology, Kağızman State Hospital, 36700 Kars, Turkey; macidov262@gmail.com; 3 Department of Cardiology, Düziçi State Hospital, 80600 Osmaniye, Turkey; abuzer.ock@gmail.com; 4 Department of Medical Pharmacology, Ataturk University, 25030 Erzurum, Turkey

**Keywords:** NSTEMI, CABG, invasive strategy, PCI, medical therapy, MACE, all-cause mortality

## Abstract

*Background and Objectives*: Patients with a prior history of coronary artery bypass grafting (CABG) who present with non-ST-segment elevation myocardial infarction (NSTEMI) represent a complex, high-risk subgroup due to advanced comorbidity burden and challenging coronary anatomy. Whether an invasive strategy offers meaningful benefit over conservative management in this population remains unclear. Therefore, this study aimed to compare long-term outcomes of percutaneous coronary intervention (PCI) versus medical therapy in NSTEMI patients with previous CABG and to identify independent predictors of major adverse cardiovascular events (MACE) and all-cause mortality. *Materials and Methods:* This retrospective cohort study included 286 NSTEMI patients with prior CABG (PCI: 112; medical therapy: 174). Baseline demographic, clinical, laboratory, and angiographic characteristics were assessed. The primary endpoint was MACE, while the secondary endpoint was all-cause mortality. Kaplan–Meier analysis evaluated survival differences, and multivariable Cox regression identified independent predictors. *Results:* During follow-up, MACE rates were comparable between PCI and medical therapy (14.3% vs. 18.9%; *p* = 0.305). All-cause mortality was likewise similar (9.8% vs. 10.3%; *p* = 0.541). Kaplan–Meier analysis showed no survival benefit with PCI (log-rank *p* = 0.334). Hypoalbuminemia independently predicted both MACE and mortality, while CKD and HF were major determinants of long-term mortality. *Conclusions:* In NSTEMI patients with prior CABG, no long-term superiority of PCI over medical therapy was observed with respect to MACE or mortality. Prognosis appears more closely linked to hypoalbuminemia, CKD, and HF than to the chosen management strategy. These findings underscore the importance of individualized and risk-adapted clinical decision-making in this complex population.

## 1. Introduction

Non-ST-segment elevation myocardial infarction (NSTEMI) represents a major clinical entity associated with substantial morbidity and mortality, particularly among patients with a high burden of comorbidities [1,2] Although early invasive management has demonstrated clinical benefit in selected subgroups within the general NSTEMI population, these findings predominantly originate from cohorts without a history of coronary artery bypass grafting (CABG) and with comparatively lower baseline risk [3,4,5]. In contrast, NSTEMI patients with prior CABG constitute a uniquely challenging group due to graft occlusion, accelerated native vessel atherosclerosis, complex coronary anatomy, advanced age, and a substantial comorbidity burden—factors that collectively distinguish them from the broader NSTEMI population.

Evidence guiding optimal management in this subset remains limited, as randomized controlled trials specifically evaluating NSTEMI patients with prior CABG are scarce.

In a cohort of patients with prior CABG presenting with NSTEMI, early revascularization was reported to be associated with a reduction in 1-year mortality [6]. Similarly, an analysis including patients with previous CABG who experienced myocardial infarction demonstrated lower in-hospital all-cause mortality and major adverse cardiovascular events (MACE) among those treated with percutaneous coronary intervention (PCI) compared with medical therapy alone [7]. However, these findings contrast with results from major randomized trials conducted in high-risk or elderly populations—such as ICTUS, RINCAL, and the Italian Elderly ACS Study—which have consistently reported that routine invasive management does not confer meaningful improvement in long-term outcomes [8,9,10]. Furthermore, a recent large-scale meta-analysis also demonstrated no significant advantage of a routine invasive strategy over conservative therapy with respect to mortality or MACE in post-CABG acute coronary syndrome populations [11].

Within this context, defining the optimal treatment approach for NSTEMI patients with a history of CABG remains one of the most contentious and underexplored areas in contemporary clinical practice. The present study aims to address this gap by comparing long-term clinical outcomes associated with percutaneous coronary intervention (PCI) versus medical therapy in NSTEMI patients with prior CABG, thereby providing a real-world perspective to inform evidence-based decision-making in this complex and high-risk population.

## 2. Materials and Methods

### 2.1. Study Design and Data Collection

**Figure 1:**
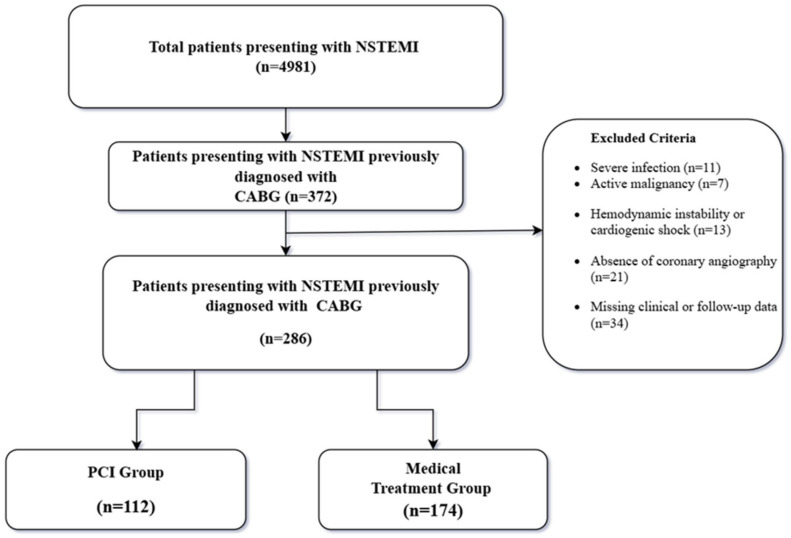
Flowchart of Study Population.

This study was conducted as a single-center, retrospective cohort analysis. A total of 4981 patients admitted to our institution with a diagnosis of NSTEMI between 2016 and 2024 were screened. Among them, 372 patients had a documented history of CABG. Patients were excluded if they had severe infection or sepsis, active malignancy, hemodynamic instability or cardiogenic shock, absence of coronary angiography, or incomplete clinical or follow-up data. After applying exclusion criteria, 286 patients were included in the final analysis (Figure 1). Based on the initial management strategy, patients were categorized into a PCI group (n = 112) and a medical therapy group (n = 174).

Comprehensive clinical information was retrieved from electronic medical records, including medical history, demographic and clinical characteristics, medication use, hematologic and biochemical parameters, echocardiographic findings, and mortality data. Coronary angiographic images were obtained from the catheterization laboratory archives. Follow-up data were systematically collected and included major clinical outcomes such as all-cause mortality, stroke or transient ischemic attack (TIA), non-fatal myocardial infarction, and hospitalization for heart failure.

### 2.2. Definition

Clinical variables and comorbid conditions were defined in accordance with current guideline recommendations. The diagnosis of NSTEMI was established based on typical ischemic symptoms accompanied by cardiac troponin elevations above the 99th percentile upper reference limit and/or new ischemic electrocardiographic changes, consistent with contemporary definitions [1]. Hypertension (HT) was defined as a previously documented diagnosis, use of antihypertensive medication, or a recorded systolic blood pressure ≥ 140 mmHg or diastolic blood pressure ≥ 90 mmHg [12]. Diabetes mellitus (DM) was defined as a prior confirmed diagnosis, use of insulin or oral antidiabetic agents, glycated hemoglobin (HbA1c) ≥ 6.5%, or fasting plasma glucose ≥ 126 mg/dL [13]. Atrial fibrillation was identified by electrocardiographic documentation or a previously recorded medical diagnosis. Chronic kidney disease (CKD) was defined by a prior diagnosis of CKD or an estimated glomerular filtration rate (eGFR) < 60 mL/min/1.73 m^2^ persisting for at least three months [14]. Heart failure (HF) was defined as a clinically confirmed diagnosis consistent with international guideline criteria [15]. A history of cerebrovascular events included clinically or radiologically confirmed ischemic stroke or transient ischemic attack.

### 2.3. Endpoints

The primary endpoint was the occurrence of major adverse cardiovascular events (MACE), defined as a composite of stroke or transient ischemic attack, non-fatal myocardial infarction, hospitalization for heart failure, and cardiovascular mortality. The secondary endpoint was long-term all-cause mortality.

### 2.4. Statistical Analysis

All statistical analyses were performed using SPSS version 27.0 (IBM Corp., Armonk, NY, USA). Continuous variables were assessed for normality using visual inspection and the Kolmogorov–Smirnov/Shapiro–Wilk tests. Normally distributed variables were presented as mean ± standard deviation (SD) and compared between groups using the independent samples *t*-test. Non-normally distributed variables were expressed as median and interquartile range (IQR) and compared using the Mann–Whitney U test. Categorical variables were summarized as percentages and compared using the χ^2^ test. A two-sided *p*-value < 0.05 was considered statistically significant. Univariable Cox proportional hazards analyses were initially performed to identify variables associated with long-term MACE and all-cause mortality. Variables with statistical significance in univariable analyses were examined for multicollinearity using Pearson correlation coefficients. Parameters with low intercorrelation were subsequently entered into a multivariable Cox regression model to determine independent predictors of outcomes. Survival analyses for all-cause mortality in the PCI and medical therapy groups were conducted using Kaplan–Meier methodology, and survival curves were compared using the log-rank test. Kaplan–Meier curves were graphically presented to illustrate differences between treatment strategies.

## 3. Results

A total of 286 patients were included in the final analysis (Figure 1). Of these, 112 patients (39.1%) were managed with an invasive strategy involving PCI, whereas 174 patients (60.9%) received conservative medical therapy. Follow-up time was 436 ± 132 days.

### 3.1. Clinical, Demographic, and Laboratory Characteristics

Baseline demographic and clinical characteristics were broadly comparable between the two groups (Table 1). The mean age did not differ significantly between patients undergoing PCI and those receiving medical therapy (69.76 ± 8.66 vs. 71.25 ± 9.55 years, *p* = 0.183). Similarly, the proportion of male patients was comparable (72.3% vs. 67.2%, *p* = 0.437). No significant differences were observed in the prevalence of hypertension, diabetes mellitus, atrial fibrillation, chronic kidney disease, cerebrovascular disease, left ventricular ejection fraction, or history of heart failure (all *p* > 0.05). The use of guideline-directed medical therapies—including aspirin, P2Y12 inhibitors, statins, ACE inhibitors/ARBs, mineralocorticoid receptor antagonists, beta-blockers, and diuretics—was likewise similar across treatment groups (all *p* > 0.05).

While most laboratory parameters were similar between groups, patients treated conservatively exhibited significantly lower serum albumin levels compared with those who underwent PCI (38.93 ± 5.24 vs. 41.11 ± 4.99 g/L, *p* = 0.001). No other laboratory values showed significant differences between groups.

### 3.2. Angiographic Findings

Angiographic characteristics are summarized in Table 2. No significant differences were observed between the two groups regarding the overall extent of coronary artery disease. The prevalence of single-vessel disease was comparable in the PCI and medical therapy groups (18.8% vs. 13.2%, *p* = 0.272), as was the prevalence of two-vessel involvement (35.7% vs. 29.9%, *p* = 0.368). Although three-vessel disease appeared more frequent in the medical therapy group (56.9% vs. 45.5%), this difference did not reach statistical significance (*p* = 0.079). Assessment of culprit lesion distribution demonstrated that native coronary arteries constituted the primary source of ischemia in both groups (PCI 68.8% vs. medical therapy 64.9%; *p* = 0.591). Culprit lesions originating from bypass grafts were also similarly distributed (PCI 31.3% vs. 35.1%; *p* = 0.591). The distribution of native culprit lesions across the RCA, LAD, and LCx territories did not differ significantly between the groups (RCA, *p* = 0.373; LAD, *p* = 0.277; LCx, *p* = 0.673). Likewise, the distribution of graft-related lesions between saphenous vein grafts (SVG) and the left internal mammary artery (LIMA) remained comparable (SVG: 25.9% vs. 27.6%, *p* = 0.858; LIMA: 5.4% vs. 7.5%, *p* = 0.647). Graft patency rates showed no statistically significant difference, with patent grafts identified in 76.8% of PCI-treated patients and 67.2% of those receiving medical therapy (*p* = 0.109).

Overall, the two groups exhibited no significant differences in the extent of coronary artery disease, the origin of the culprit lesion, or graft patency status.

### 3.3. Clinical Outcomes

The duration of follow-up was comparable between the PCI and medical therapy groups (448 ± 128 vs. 429 ± 134 days, *p* = 0.206). There were no significant differences in individual components of major adverse cardiovascular events (MACE), including stroke/transient ischemic attack, non-fatal myocardial infarction, hospitalization for heart failure, or cardiovascular mortality. The overall incidence of MACE was similar between the groups (PCI: 14.3% vs. medical therapy: 18.9%; *p* = 0.305).

Long-term all-cause mortality was likewise comparable, occurring in 7.1% of patients in the PCI group and 9.2% of those in the medical therapy group (*p* = 0.541) (Table 3).

**Figure 2:**
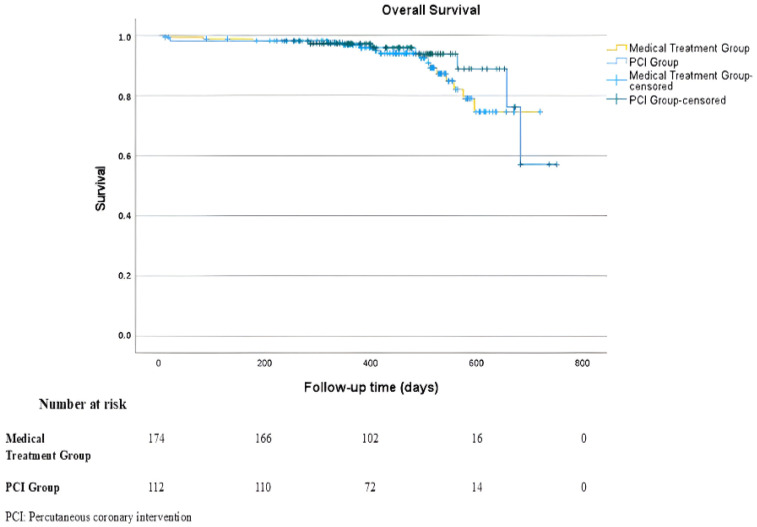
Kaplan–Meier Survival Curve for All-Cause Mortality.

Kaplan–Meier survival analysis demonstrated no significant difference in overall survival between the two treatment strategies (log-rank *p* = 0.334) (Figure 2).

### 3.4. Predictors of MACE

In the univariable Cox analysis (Table 4), several clinical variables were significantly associated with the development of MACE. Lower serum albumin level (HR = 0.88, *p* = 0.001), CKD (HR = 2.19, *p* = 0.010), HF (HR = 2.06, *p* = 0.012), DM (HR = 2.39, *p* = 0.005), and higher HbA1c levels (HR = 1.29, *p* = 0.018) were all identified as significant correlates of adverse outcomes.

In the -multivariable Cox regression model, low albumin (HR = 0.92, *p* = 0.006) and CKD (HR = 2.51, *p* = 0.006) remained independent predictors of MACE.

### 3.5. Predictors of Long-Term Mortality

In the univariable Cox analysis (Table 5), several clinical variables were significantly associated with long-term all-cause mortality. Lower serum albumin level (HR = 0.77, *p* < 0.001), CKD (HR = 6.63, *p* < 0.001), HF (HR = 3.05, *p* = 0.008), and DM (HR = 5.63, *p* = 0.001) were all identified as significant predictors of mortality.

In the multivariable Cox regression model, serum albumin (HR = 0.82, *p* < 0.001), CKD (HR = 6.09, *p* < 0.001), and HF (HR = 2.62, *p* = 0.030) remained independent determinants of long-term all-cause mortality.

## 4. Discussion

This study compared long-term clinical outcomes associated with PCI versus medical management in patients presenting with NSTEMI who had a prior history of CABG. Our findings indicate that PCI did not confer a significant advantage over conservative medical therapy with respect to either major adverse cardiovascular events (MACE) or all-cause mortality. These results align with and further reinforce the growing body of evidence suggesting that routine invasive management offers limited clinical benefit in this uniquely high-risk population [11].

Patients with a history of CABG represent a small yet clinically critical subset of the overall NSTEMI population [11,16,17]. Real-world data consistently show that individuals with prior CABG are referred for invasive evaluation at markedly lower rates compared with CABG-naïve NSTEMI patients. Several observational studies have demonstrated that invasive management is undertaken less frequently than conservative therapy in this group [11,18], reflecting the complexity of post-CABG coronary anatomy, heightened procedural risk, and the perceived limited incremental benefit of PCI. These trends suggest that clinicians often adopt a more selective and individualized approach when considering invasive strategies for post-CABG NSTEMI patients.

In our cohort, 39% of patients underwent PCI, whereas 61% were managed medically, a distribution closely mirroring contemporary real-world practice. This concordance supports the external validity of our study and reinforces that our findings reflect treatment patterns observed in routine clinical care.

A review of the contemporary literature demonstrates that the clinical benefit of a routine invasive strategy in NSTEMI patients with prior CABG is consistently limited. In a cohort of patients with known prior CABG presenting with NSTEMI, revascularization performed within 14 days of admission was reported to be associated with a reduction in 1-year mortality [6]. Similarly, an analysis including patients with previous CABG who experienced myocardial infarction demonstrated lower in-hospital all-cause mortality and MACE among those treated with PCI compared with medical therapy alone [7]. In line with these findings, another study reported that PCI was associated with a lower risk of in-hospital mortality in patients with prior CABG, although rates of reinfarction were comparable to those observed with medical management [19].

In contrast, large-scale analyses indicate that invasive management does not significantly reduce mortality or MACE in this population [11,17]. Importantly, landmark randomized trials that established the benefits of early invasive therapy in the general NSTEMI population—such as FRISC-II and TACTICS-TIMI 18 [3,4]—explicitly excluded patients with prior CABG. As a result, the favorable outcomes observed in these trials cannot be readily extrapolated to this anatomically complex and high-risk subgroup.

Consequently, the available evidence is largely derived from real-world observational studies and targeted subgroup analyses. Trials such as ICTUS, RINCAL, and the Italian Elderly ACS Study, which focused on older, frail, and comorbidity-burdened populations, consistently demonstrated no superiority of routine invasive management over conservative therapy [8,9,10] Similarly, the CABG-ACS study—which specifically evaluated patients with prior CABG—found that an invasive approach did not yield meaningful improvements in mortality or MACE compared with medical management [18]. These findings have been further reinforced by robust large-scale meta-analyses [11].

Large observational studies provide additional support for this pattern. Gurfinkel et al. reported comparable rates of death, recurrent ischemia, and myocardial infarction between medically treated and revascularized CABG patients presenting with ACS [20]. In line with these results, Asrar-ul-Haq et al. demonstrated no difference in rehospitalization, recurrent UA/ACS, or mortality at one-year follow-up between invasively and conservatively treated patients [21]. Moreover, a subgroup analysis of the ACUITY trial showed significantly higher 30-day and 1-year MACE rates in ACS patients with previous CABG who underwent PCI compared with those treated conservatively [22].

Consistent with the existing literature, our study also demonstrated that an invasive treatment strategy did not confer a significant advantage in terms of clinical outcomes among NSTEMI patients with prior CABG. This observation suggests that the unique clinical and anatomical characteristics of post-CABG patients may attenuate the potential benefit of invasive approaches. In our analysis, the higher tendency toward three-vessel disease and the increased frequency of graft-related culprit lesions in the medically managed group underscore the complexity of coronary anatomy in this population. Factors such as graft occlusion, diffuse native vessel disease, surgically altered coronary architecture, and the technical challenges of PCI in post-CABG anatomy may collectively diminish the anticipated benefit of revascularization [17].

These findings align closely with current evidence indicating that, in the post-CABG population, treatment decisions should be individualized and guided by patient-specific risk assessment rather than routine application of an invasive strategy.

One of the most compelling findings of our study is that clinical outcomes in this population were predominantly driven by systemic comorbidities—particularly hypoalbuminemia, CKD, and HF—rather than by the choice of treatment strategy. Low serum albumin has been repeatedly linked to malnutrition, chronic inflammation, and diminished metabolic reserve [23,24,25]. In elderly patients with multiple comorbidities, hypoalbuminemia correlates strongly with frailty and is consistently associated with an increased risk of cardiovascular events and mortality [26,27,28]. CKD remains one of the strongest determinants of mortality in ACS populations due to its association with endothelial dysfunction, chronic inflammation, and accelerated atherosclerosis [29,30]. Similarly, HF significantly elevates the risk of mortality through mechanisms involving hemodynamic compromise, neurohormonal activation, and reduced cardiac reserve [31,32,33].

In our multivariable analyses, albumin and CKD emerged as independent predictors of MACE, whereas albumin, CKD, and HF were independent determinants of long-term all-cause mortality. These results underscore that clinical trajectories in post-CABG NSTEMI patients are shaped not only by coronary anatomical features or graft status but also, and perhaps more profoundly, by the systemic burden of comorbidity. Indeed, a growing body of evidence highlights that event risk in CABG patients is strongly influenced by global risk factors that reflect overall physiological vulnerability, in addition to graft-related pathophysiology [17,34].

When these findings are considered collectively, they support the notion that therapeutic decision-making in NSTEMI patients with a history of CABG should be individualized rather than routine or protocol-driven. Our results suggest that long-term clinical trajectories in this population are determined less by the choice of an invasive strategy and more by systemic factors such as nutritional status, frailty, inflammation, and coexisting comorbidities. This reinforces the growing body of evidence indicating that the long-term benefit of invasive management is limited in post-CABG NSTEMI patients and that global physiological vulnerability may play a more decisive role in shaping outcomes than coronary anatomical features alone.

### Limitations

This study has several limitations that warrant consideration. First, its retrospective observational design precludes the establishment of causal relationships and cannot fully eliminate the possibility of residual confounding. Moreover, the choice of treatment strategy was influenced by clinician judgment, introducing potential selection bias. The lack of detailed procedural information—such as PCI technical success, stent type, and lesion morphology—may have limited our ability to fully characterize the true impact of invasive management. Furthermore, lower baseline albumin levels in the medical therapy group may reflect a potential selection bias, whereby clinically more frail patients were preferentially directed toward conservative management based on physician judgment. The single-center nature of the study may restrict the generalizability of the findings to broader populations. Finally, the relatively limited sample size should be considered, as it may have reduced the statistical power required to detect small but clinically meaningful differences in survival. Despite these limitations, the study provides valuable real-world data regarding long-term outcomes of PCI versus medical therapy in NSTEMI patients with prior CABG.

## 5. Conclusions

This study demonstrates that, among NSTEMI patients with a history of CABG, PCI does not confer a significant advantage over medical therapy in reducing either MACE or all-cause mortality. Taken together, these results underscore that routine invasive management should not be the default approach in this population. Instead, therapeutic decisions should be selective, individualized, and guided by a comprehensive assessment of each patient’s comorbidity profile and overall risk status.

## Figures and Tables

**Table 1:** Baseline Demographic, Clinical and Laboratory Characteristics According to Management Strategy.

Variables	PCI Group (n = 112)	Medical Therapy Group (n = 174)	*p* Value
Demographics			
Age (years)	69.76 ± 8.66	71.25 ± 9.55	0.183
Sex (male, %)	81 (72.3%)	117 (67.2%)	0.437
Medical History			
HT (n, %)	78 (69.6%)	133 (76.4%)	0.255
DM (n, %)	54 (48.2%)	89 (51.1%)	0.716
AF (n, %)	15 (13.4%)	16 (8.6%)	0.277
CKD (n, %)	18 (16.1%)	31 (17.8%)	0.820
CVE (n, %)	7 (6.2%)	18 (10.3%)	0.326
HF (n, %)	35 (31.2%)	62 (35.6%)	0.525
Ejection Fraction (%)	49.0 ± 6.8	47.5 ± 6.3	0.070
Medications			
ASA (n, %)	106 (94.6%)	161 (92.5%)	0.647
P2Y12is	17 (15.2%)	22(12.6%)	0.510
Statins (n, %)	97 (86.6%)	154 (88.5%)	0.769
ACEI/ARB (n, %)	76 (67.9%)	140 (80.5%)	0.280
MRA (n, %)	27 (24.1%)	48 (27.5%)	0.490
BB (n, %)	93 (83.0%)	137 (78.7%)	0.458
Diuretics (n, %)	28 (25.0%)	51 (29.3%)	0.468
Laboratory Parameters			
WBC (10^9^/L)	8.58 ± 2.37	8.38 ± 2.25	0.471
Hemoglobin (g/dL)	12.33 ± 1.39	12.52 ± 1.65	0.306
Neutrophil count (10^9^/L)	6.03 ± 2.25	5.67 ± 2.07	0.158
Platelet count (10^9^/L)	218.44 ± 54.78	229.73 ± 57.24	0.450
Lymphocyte count (10^9^/L)	1.83 ± 0.61	2.14 ± 0.59	0.309
CRP (mg/L)	15.92 ± 8.39	17.47 ± 6.77	0.474
Albumin (g/L)	41.11 ± 4.99	38.93 ± 5.24	0.001
Creatinine (mg/dL)	1.20 ± 0.38	1.17 ± 0.37	0.548
Glucose (mg/dL)	157.51 ± 44.12	149.72 ± 36.48	0.106
Troponin (ng/L)	620 ± 234	590 ± 302	0.126
HbA1c (%)	7.33 ± 1.32	7.23 ± 1.34	0.556
LDL (mg/dL)	116.71 ± 36.29	115.11 ± 33.42	0.703
HDL (mg/dL)	40.26 ± 9.78	41.41 ± 9.58	0.324
Triglyceride (mg/dL)	170.15 ± 58.98	160.72 ± 55.65	0.173

Abbreviations: HT, hypertension; DM, diabetes mellitus; AF, atrial fibrillation; CKD, chronic kidney disease; CVE, cerebrovascular event; HF, heart failure; EF, Ejection Fraction; ASA, acetylsalicylic acid; P2Y12is: P2Y12 receptor inhibitors; ACEI, angiotensin-converting enzyme inhibitor; ARB, angiotensin receptor blocker; MRA, mineralocorticoid receptor antagonist; BB, beta-blocker; WBC: white blood cell; CRP, C-reactive protein, HDL: High-Density Lipoprotein, LDL: Low-Density Lipoprotein.

**Table 2:** Angiographic Characteristics According to Management Strategy.

Variables	PCI Group (n = 112)	Medical Therapy Group (n = 174)	*p* Value
Extent of CAD			
1-vessel disease	21 (18.8%)	23 (13.2%)	0.272
2-vessel disease	40 (35.7%)	52 (29.9%)	0.368
3-vessel disease	51 (45.5%)	99 (56.9%)	0.079
Culprit Vessel			
Native vessel	77 (68.8%)	113 (64.9%)	0.591
Graft (SVG/LIMA)	35 (31.3%)	61 (35.1%)	0.591
Native Culprit Distribution			
RCA	23 (20.5%)	45 (25.9%)	0.373
LAD	33 (29.5%)	40 (23.0%)	0.277
LCx	21 (18.8%)	28 (16.1%)	0.673
Graft Culprit Distribution			
SVG	29 (25.9%)	48 (27.6%)	0.858
LIMA	6 (5.4%)	13 (7.5%)	0.647
Graft Status			
Patent grafts	86 (76.8%)	117 (67.2%)	0.109
Occluded grafts	26 (23.2%)	57 (32.8%)	0.109

Abbreviations: SVG, saphenous vein graft; LIMA, left internal mammary artery; RCA, right coronary artery; LAD, left anterior descending artery; LCx, left circumflex artery.

**Table 3:** Clinical Outcomes According to Management Strategy.

Outcomes	PCI Group (n = 112)	Medical Therapy Group (n = 174)	*p*-Value
Stroke/TIA (n,%)	3 (2.7%)	5 (2.9%)	0.922
Non-fatal MI (n,%)	7 (6.3%)	12 (6.9%)	0.830
HF hospitalization (n,%)	7 (6.3%)	13 (7.5%)	0.693
CV Mortality (n,%)	5 (4.5%)	9 (5.2%)	0.786
Totally MACE (n,%)	16 (14.3%)	33 (18.9%)	0.305
All-cause mortality (n,%)	8 (7.1%)	16 (9.2%)	0.541
Follow-up time (day)	448 ± 128	429 ± 134	0.205

Abbreviations: TIA, transient ischemic attack; MI, myocardial infarction; MACE, major adverse cardiovascular events; HF, heart failure; CV, cardiovascular.

**Table 4:** Cox Regression Analysis According to MACE Development Status.

Variables	Univariate HR, 95%CI	*p* Value	Multivariate HR, 95% CI	*p* Value
Albumin (g/L)	0.88 (0.83–0.94)	0.001	0.92 (0.87–0.98)	0.006
CKD	2.19 (1.20–3.99)	0.010	2.51 (1.31–4.80)	0.006
HF	2.06 (1.17–3.61)	0.012	1.69 (0.93–3.05)	0.084
DM	2.39 (1.30–4.42)	0.005	1.72 (0.90–3.28)	0.100
HbA1c (%)	1.29 (1.04–1.58)	0.018	1.22 (0.98–1.52)	0.075

Abbreviations: CKD, chronic kidney disease; HF, heart failure; DM, diabetes mellitus; HR, hazard ratio; CI, confidence interval.

**Table 5:** Cox Regression Analysis According to All-Cause Mortality Development Status.

Variables	Univariate HR, 95% CI	*p* Value	Multivariate HR, 95% CI	*p* Value
Albumin (g/L)	0.77 (0.70–0.84)	<0.001	0.82 (0.74–0.91)	<0.001
CKD	6.63 (2.93–15.00)	<0.001	6.09 (2.34–15.88)	<0.001
HF	3.05 (1.33–6.99)	0.008	2.62 (1.10–6.26)	0.030
DM	5.63 (1.89–16.74)	0.001	2.23 (0.69–7.15)	0.179

Abbreviations: CKD, chronic kidney disease; HF, heart failure; DM, diabetes mellitus; HR, hazard ratio; CI, confidence interval.

## Data Availability

The data presented in this study are available on request from the corresponding author. Data are not publicly available due to ethical and patient privacy considerations.

## Abbreviations

The following abbreviations are used in this manuscript:

CABG	Coronary Artery Bypass Grafting
PCI	Percutaneous Coronary Intervention
NSTEMI	Non-ST Segment Elevation Myocardial Infarction
ACS	Acute Coronary Syndrome
UA	Unstable Angina
ASA	Acetylsalicylic Acid
MACE	Major Adverse Cardiovascular Events
HF	Heart Failure
CKD	Chronic Kidney Disease
GFR	Glomerular Filtration Rate
LAD	Left Anterior Descending Artery
RCA	Right Coronary Artery
LCx	Left Circumflex Artery
LIMA	Left Internal Mammary Artery
SVG	Saphenous Vein Graft
HbA1c	Glycated Hemoglobin
ACE	Angiotensin-Converting Enzyme
ARB	Angiotensin Receptor Blocker
TIA	Transient Ischemic Attack
